# Development and evaluation of an indirect enzyme-linked immunosorbent assay for serological detection of Schmallenberg virus antibodies in ruminants using whole virus antigen

**DOI:** 10.1186/s13028-014-0071-1

**Published:** 2014-12-05

**Authors:** Katarina Näslund, Gunilla Blomqvist, Caroline Vernersson, Stéphan Zientara, Emmanuel Bréard, Jean F Valarcher

**Affiliations:** Department of Virology, Immunobiology and Parasitology, National Veterinary Institute, SE-75189 Uppsala, Sweden; Virology Unit, French Agency for Food Environmental and Occupational Health Safety, F-94703 Maisons-Alfort, France

**Keywords:** Indirect ELISA, Whole virus antigen, Schmallenberg virus, Antibody detection, Cattle, Sheep, Goat

## Abstract

**Background:**

In late 2011, a new *Orthobunyavirus* of the Simbu serogroup named Schmallenberg virus (SBV) emerged in continental Europe. The virus is transmitted by hematophagous arthropods, with the *Culicoides* species as, so far known, main vectors. Infection with the virus can cause clinical signs in adult ruminants including diarrhea, fever and reduced milk production. Transplacental infection of the developing fetus can lead to malformations of varying severity. To assess seroprevalence of SBV in Sweden an indirect enzyme-linked immunosorbent assay (ELISA) was established in connection with the surveys. Here, we describe the development and evaluation of the indirect ELISA, based on whole virus as the coating antigen and a monoclonal antibody for the detection of antibodies to SBV in ruminant sera. The evaluation includes comparison between the in-house ELISA, virus neutralization test and an indirect commercial ELISA.

**Results:**

The optimal working dilutions of antigens and conjugate were estimated with checkerboard titrations. Comparative studies, including ROC analyses, were used for the selection of an optimal cut-off (S/P value = sample value as percentage of positive control value). With an estimated S/P value of 15% the whole virus ELISA showed a specificity of 100% and a sensitivity of 99.19% compared to virus neutralization test (VNT) and with a good consistency as shown in reproducibility and variability experiments. Furthermore, the comparison of our whole virus indirect ELISA to an indirect ELISA with a SBV nucleoprotein antigen, demonstrated a higher sensitivity of our test.

**Conclusion:**

The indirect whole virus ELISA described in this paper is a readily available test for serological analysis of SBV antibodies. Since this in-house ELISA demonstrates a specificity and sensitivity comparable to virus neutralization test and also shows a higher sensitivity compared to commercially available indirect ELISA, it is a useful alternative for surveillance and screening purposes of SBV.

## Background

Since Schmallenberg virus (SBV) was identified in October 2011 [1] cases of SBV infection have been reported in ruminants in most European countries [2] and, as of late 2012, also in Sweden [3]. In cattle, clinical signs include loss of appetite, fever, diarrhea and decreased milk production [4-6]. In addition, if SBV infection of fetuses occurs during a vulnerable stage in pregnancy, the SBV can induce neurological disorders in newborns and may lead to congenital malformations with intra-uterine or neonatal death [7-11].

The Schmallenberg virus is a member of the genus *Orthobunyavirus* within the family *Bunyaviridae* and related to the *Simbu* serogroup [1]. The virus genome consists of 3 segments of negative-sense single-stranded RNA: the L (large), M (medium) and S (small) segments [12]. The enveloped virus particle has a diameter of approximately 100 nm [13] and is composed of 4 structural proteins: two surface glycoproteins, the Gn and Gc, the polymerase protein (L) and the nucleoprotein (N). Results of full-genome and serologic investigations indicate that SBV belongs to the species *Sathuperi* virus and is not a reassortant but rather likely one of the ancestors of *Shamonda* virus [14].

In the spring of 2012, before the vector season started, several serosurveys were performed in Sweden [3]. At that time, only one commercial indirect ELISA based on a recombinant SBV nucleocapside protein antigen was available [15]. It was found that this test sometimes gave unspecific results according to virus neutralizing test performed at our laboratory and at Animal Health Laboratory at ANSES. Also, the virus neutralization test developed at our laboratory did not have the capacity for more extensive surveys. Since the surveys were planned to include both sera and bulk milk it was desirable to establish an ELISA potentially useful for both sera and milk which also was rapid and sensitive enough for mass screening.

The present study describes the establishment and evaluation of an indirect ELISA for the detection of antibodies to SBV in cattle, sheep and goat sera.

## Methods

### Virus

The Schmallenberg virus isolate BH80/11-4, kindly provided by the Friedrich-Loeffler-Institut, Germany, was used for the ELISA antigen preparation and in virus neutralization test (VNT) [1,14]. After an initial propagation in BHK-21 cells, the virus was passaged on Vero cells cultivated in Eagle’s minimal essential medium (EMEM) complete (SVA, Sweden) with 2% fetal bovine serum (FBS). A master seed stock of 10^4.25^ TCID_50_/ml was prepared, aliquoted and stored in −80°C until used.

### Serum samples

SBV positive and negative ruminant sera, as confirmed by virus neutralizing test (VNT), were used for determining cut-off values, specificity and sensitivity of the in-house ELISA and for comparative studies between the in-house ELISA, VNT and a commercial ELISA.

The sera included three hundred negative sera collected from Swedish holdings before any introduction of SBV to Sweden was confirmed as well as positive sera from naturally infected animals, including 64 bovine, 48 ovine and 11 caprine sera from France, The Netherlands, Finland and Sweden (see Table 1).

**Table 1:** **Origin of virus neutralization test positive and negative Schmallenberg virus sera used**

**Origin of sera**	**Bovine**		**Ovine**		**Caprine**	
	**Pos**	**Neg**	**Pos**	**Neg**	**Pos**	**Neg**
France, Animal Health Laboratory at ANSES	29		21			
The Netherlands, Animal Health Service (GD)			17			
Finland, Finnish Food Safety Authory, (EVIRA)	9		1			
Sweden, National Veterinary Institute, (SVA)	23	^1^100	9	^2^100	11	^3^100
Total n:o of sera	64	100	48	100	11	100

^1^collected in 2005.

^2^collected in 2011.

^3^collected in 1997.

### SBV neutralizing antibody assay

Serum samples were analyzed for neutralizing antibodies in a virus neutralizing test (VNT) designed for SBV at our laboratory. The virus isolate used was BH80/11-4, and passaged in BHK-21 cells cultivated in Eagle’s minimal essential medium (EMEM) complete (SVA, Sweden) with 2% FBS. Before analyzing, the sera were heated for 30 min at 56°C. The VNT was performed in 96-well microtitreplates in which sera were 2-fold diluted in EMEM in volumes of 50 μl in duplicate starting from 1:2 up to 1:256. Between 30 and 300 TCID_50_ of virus in a volume of 50 μl per well was then added to the microtitreplates with the exception of the first row with 1:2 serum dilutions where only medium was added (serum controls). After preincubation at 37°C for 1 h approximately 20000 cells in a volume of 50 μl in EMEM supplemented with 20% FBS were added to each well. The plates were then incubated for 3–4 days at 37°C under 5% CO_2_. After incubation, the plates were examined in a light microscope for the presence of virus specific cytopathogenic effects (cpe). The neutralizing titer of a serum was determined as the highest dilution in which the cell monolayer was intact. In each run of the VNT, positive and negative control sera were included.

### Commercial indirect ELISA for detection of SBV antibodies

A commercial indirect ELISA based on a recombinant SBV nucleocapside protein antigen and a multi-species conjugate [15] was used for comparative purposes. The analysis and the interpretation of the results were performed in accordance to the protocol. The optical density (OD) of a sample tested (S) was compared with the OD of the positive control (PC) to give an S/P percentage value (OD_sample)_/OD_positive control_ × 100). A S/P value of >60% was considered as positive.

### Preparation of ELISA antigens

SBV was cultivated in Vero cells for the preparation of virus antigen. Non infected Vero cells were used for the preparation of control antigen.

Vero cells were grown at 37°C to 80-90% confluence in 162 cm^2^ tissue culture bottles (Costar, Corning Inc., USA). The medium was removed and the cell monolayer was rinsed with phosphate-buffered saline (PBS) and then inoculated with 10 ml of virus diluted 1:10 in EMEM without FBS. After one hour of adsorption at 37°C, 40 ml of EMEM with 2% FBS was added to the virus infected cells. The cultures were then incubated at 37°C under daily observation. When a cytopathogenic effect of 80-90% was seen, usually after 2 days, the cultures were frozen at −80°C. Non-infected Vero cells grown and handled under similar conditions were used for the preparation of control antigen. The cell bottles were freeze-thawed consecutively two times and the material was centrifuged at 1500 x g for 15 min. The pelleted cell debris were dissolved in 1/100 of the starting volume in PBS and stored in −80°C until used. The supernatants were precipitated and concentrated with 10% polyethylene glycol (PEG) as described by Simard *et al.* [16] with minor modifications. Briefly, the supernatants were mixed with 30% (w/v) PEG 8000 (Fisher Scientific) in 0.4 M NaCl at a ratio of 2:1 and stirred for 1 h at 4°C. The precipitates were pelleted by centrifugation at 1500 x g for 20 min and thereafter dissolved in 1/100 of the original volume in PBS, dialyzed against Super Q water over night and finally against PBS for 4 h with the use of a dialysis membrane with 12–14 kDalton cut off (Spectra/Por® 2, VWR). To solubilise and inactivate virus, the PEG precipitated concentrates of virus and the cell bound virus of the cell debris fractions were treated by adding Triton x-100 (Merck) to a final concentration of 0.1%, 0.5 and 1% (v/v). The solutions were then sonified in two cycles of 5 min in an ultrasonic water bath (Bandelin Sonorex) and incubated with agitation at room temperature for 15 min between the sonifying steps, clarified by centrifugation for 15 min at 1500 × g and then stored in −80°C until used. The different concentrations of Triton x-100 of the virus inactivation step were evaluated by three passages in Vero cells. Before inoculation the Triton x-100 was removed due to its cell toxicity by gel filtration on Sephadex G-25 GE-Healthcare Uppsala Sweden) in PBS [17].

### Optimization of ELISA

Checkerboard titrations were performed to find optimal working dilutions of antigens and conjugate. Three variants of virus and control antigens were tested; the antigen concentrated by PEG saturation (A), antigen from the cell debris fraction (B) and a combination of PEG precipitatedted antigen and cell debris antigen (A + B). Bovine sera with a VNT titer 1:128 and <1:4 was used as positive and negative controls. A HRP conjugated monoclonal antibody anti-bovine IgG_1_ also binding to ovine and caprine IgG (Boehringer Ingelheim Svanova, Sweden) was used as conjugate. The antigens were initially titrated in two-fold dilutions from 1:1000 to 8000, and thereafter evaluated in the dilution range of 1:1500 to 1:4000. The sera were diluted 1:100 and the conjugate was diluted 1:5000, 1:10 000, 1:20 000 and 1:40 000.

### Western blot analysis

The antigen consisting of the A + B combination were separated on 10% SDS-PAGE and transferred onto a 0.45 μm nitrocellulose membrane (Bio-Rad) at 100 V for 1 h with a Criterion™ Blotter (Bio-Rad). The membrane was blocked with 5% skimmed milk powder in Tris-buffered saline pH 7.6 with 0.05% Tween-20 (TBS-T) and cut into stripes. The SBV proteins on the blot were detected by SBV positive sheep and cattle sera. Sera from uninfected animals were used as controls. All sera were diluted 1:100 in TBS-T and incubated for 1 h at room temperature (RT). The stripes were washed with TBS-T and then incubated with a HRP conjugated mouse anti-bovine IgG_1_ (Boehringer Ingelheim Svanova, Sweden) diluted 1:10 000 in TBS-T. After one hour incubation at RT the strips were washed and the bound secondary antibodies were detected by using ECL substrate and Hyper film ECL (Amersham/GE Healthcare).

### Final ELISA protocol

According to the results from checkerboard titrations of the different preparations of ELISA reagents the following basic protocol was implemented:

Microtitreplates (Polysorp; Nunc, Denmark) where coated with virus antigen and control antigen diluted 1:3000 in 0.05 M carbonate buffer, pH 9.6 and incubated overnight at 4°C. The plates were washed three times with 0.01 M PBS, pH 7.4 containing 0.05% Tween-20 (PBST). Serum samples diluted 1:100 in PBST with 3% FBS were added in an antigen and a control antigen coated well. After incubation for one hour at 37°C the plates were washed three times with PBST and the conjugate at a 1:20 000 dilution in PBST was added. The plates were then incubated for one hour at 37°C. After washing four times in PBST a TMB (3, 3’, 5, 5’-tetramethylbenzidine) substrate solution was added and incubated for 10 min at room temperature thereafter the reaction was stopped by adding 2 M H_2_SO_4_. The optical density (OD) was measured at 450 nm with a Multiskan EX microplate photometer (Thermo Labsystems). Positive, low positive and negative control sera were included on each plate for the evaluation of each assay. The OD value of each sample and control sera was calculated as the difference between the OD values of the virus antigen well and the control antigen well (net OD values). The results of the serum samples were then expressed as percentage of the positive control serum (S/P%).

### Determining the cut-off values and calculation of sensitivity and specificity

Three hundred VNT negative and 123 positive cattle, sheep and goat sera (see Table 1) were analyzed in a two-graph receiver operating characteristic (TG-ROC) curve to determine the optimal cut-off value and to calculate the sensitivity and the specificity.

### Inter and intra-assay variation

Four positive bovine sera were tested in 12 replicates to evaluate the inter- and intra-assay variation of the in-house ELISA. The sera were tested on five different occasions and the coefficient of variation (CV) was calculated between plates for the inter-assay variation (reproducibility) and within the plates for the intra-assay variation (variability).

### Comparison between in-house ELISA, VNT and a commercial ELISA

The relative specificity and sensitivity of the in-house ELISA and a commercial ELISA [15] were compared to VNT with the use of 112 positive and 200 negative cattle and sheep sera (see Table 2). In addition, four positive bovine sera were used undiluted and serially two-fold diluted for comparison of sensitivity between the in-house ELISA and the commercial ELISA versus VNT (Table 3).

**Table 2:** **Relative specificity and sensitivity of in-house Schmallenberg virus (SBV) ELISA and a commercial SBV ELISA compared to virus neutralization test (VNT)**

**VNT**	**In-house SBV ELISA**				**Commercial SBV ELISA**			
	**Cattle**		**Sheep**		**Cattle**		**Sheep**	
	**Pos**	**Neg**	**Pos**	**Neg**	**Pos**	**Neg**	**Pos**	**Neg**
Pos	64		47	1	59	5	39	9
Neg		100		100		100		100
Specificity	100% (100/100)		100% (100/100)		100% (100/100)		100% (100/100)	
Sensitivity	100% (64/64)		97.9% (47/48)		92.2% (59/64)		81.2% (39/48)	

### Data analysis

MedCalc® v2.3.0 software was used for the TG-ROC analysis and dot plot graph.

## Results

### Antigen preparation and optimization of the in-house ELISA

After cultivating SBV on Vero cells the cultures were freeze-thawed and then centrifuged to separate the cell debris from the cultivating material. Virus in the supernatant was concentrated by PEG precipitation. Both the pelleted cell debris fraction and the PEG concentrated virus were then treated with Triton x-100 at three different concentrations of 0.1%, 0.5%, 1.0%, to explore the concentration required for an optimal solubilization and virus inactivation. The preparations were inoculated on to Vero cells to detect any presence of infectious virus by its CPE. Virus prevalence was found already at the first passage when a concentration of 0.1% of Triton x-100 was used. At a Triton x-100 concentration of 0.5% virus could be demonstrated after 2 passages on cell cultures. When a Triton x-100 concentration of 1.0% was used no virus could be detected even after three passages on cell cultures.

The virus content of the cell debris fraction and the PEG concentrated virus was preliminary evaluated in an ELISA by serially dilutions. Both fractions were treated with 1.0% Triton x-100, in addition the cell debris fraction was centrifuged to remove residual cell debris, before the evaluation. Since it was found that the amount of cell bound virus was significant, three different preparations were finally evaluated, one from the PEG concentrated virus (A), one from the original cell debris (B) and a combination of both (A + B).

**Figure 1:**
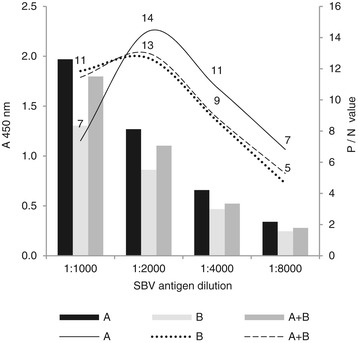
**Serially dilutions of three variants of Schmallenberg virus ELISA antigen.** A: PEG precipitated antigen, B: cell debris derived antigen and A + B: a combination of A and B tested with positive and negative sera diluted 1:100 and conjugate diluted 1:20 000. Bars shows optical density (OD) for positive serum, lines shows ratio between positive and negative sera (P/N value).

**Figure 2:**
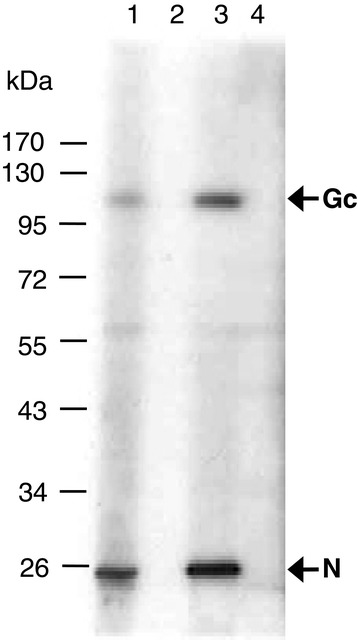
**Identification of nucleoprotein (N) and glycoprotein (Gc) of the in-house ELISA antigen.** Western blot analysis of the final ELISA antigen (A + B) was performed using sera from naturally SBV infected sheep (lane 1) and cattle (lane 3). Sera from uninfected sheep and cattle were used as controls (lane 2 and 4).

Checkerboard titrations were performed to select the optimal dilution and preparation of the three different antigen variants and the optimal dilution of conjugate. Figure 1, shows the ratio between the positive and negative test sera (P/N value) and the OD values of a positive serum against the different antigen dilutions. A maximum P/N value for all three antigen preparations was obtained with an antigen dilution of 1:2000. From the comparative checkerboard titrations only a slight difference in P/N values between the PEG concentrated virus (A) and the combined antigen (A + B), could be found. By using the combined antigen A + B, the antigen recovery was increased with approximately 75%. Further titrations of the combined antigen preparation were carried out and an antigen dilution of 1:3000 and a conjugate dilution of 1:20 000 was found to provide the lowest background while maintaining a maximum P/N value. Western blot analysis of the ELISA antigen with the use of sera from SBV naturally infected and non infected cattle and sheep revealed the presence of both nucleocapside protein (N) and the glycoprotein Gc (Figure 2).

The reproducibility of the antigen preparation method was confirmed by the comparison of three separately manufactured batches (data not shown).

### Determining the cut-off values and the relative sensitivity and specificity

**Figure 3:**
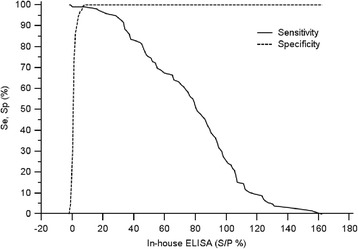
**TG-ROC curve.** The curves show the sensitivity and specificity relative to VNT at different cut-off values (S/P%) of the in-house ELISA. All of three hundred VNT negative and 123 positive bovine, caprine and ovine sera (see Table 1) were used for analysis. Within the range of S/P 8% to 15% the analysis gives a sensitivity of 99.19% and a specificity of 100%.

**Figure 4:**
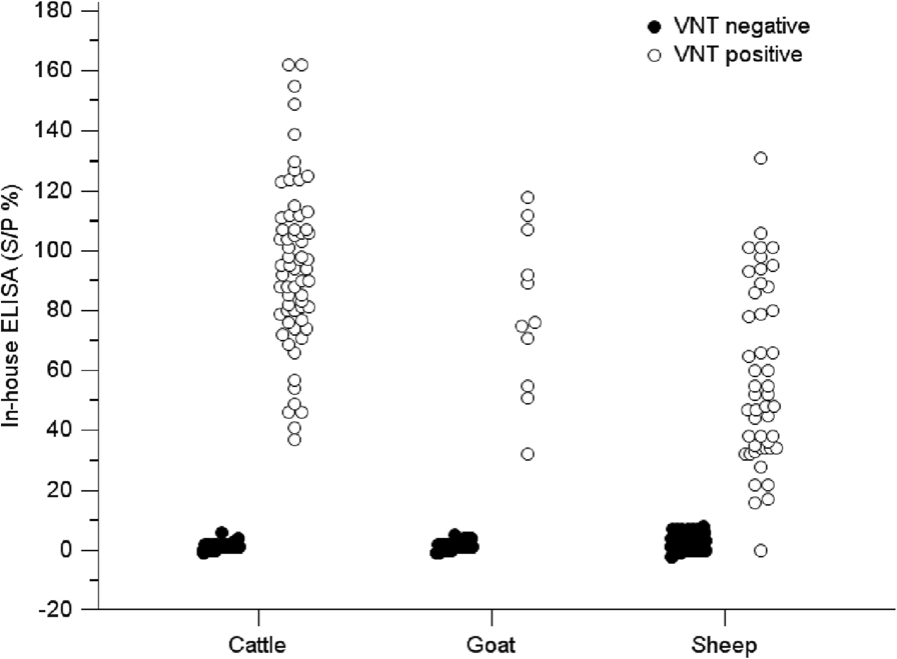
**The dot plot graph illustrates the distribution of ELISA S/P% values relative to virus neutralizing test (VNT) antibody titers.** Three hundred VNT negative and 125 VNT positive bovine, caprine and ovine sera (see Table 1) were included in the analysis. The S/P values of the negative bovine sera varied from −1 to 6 and the positive sera from 37 to 162. The S/P values of the negative caprine sera varied from −2 to 5 and the positive sera from 32 to 118. The S/P values of negative ovine sera ranged from −2 to 8 and the positive sera from 16 to 131. One VNT positive ovine sera got an S/P value of 0.

To determine the cut-off value and to calculate the relative sensitivity and specificity a TG-ROC analysis was performed with the use of all VNT positive and VNT negative sera (see Table 1). It was found that a S/P value ranging from 8 to 15% gave a sensitivity of 99.19% and a specificity of 100% (Figure 3). The distribution of the S/P values of each group is shown in Figure 4. The cut-off value was further validated by comparing the analysis results of the in-house ELISA and VNT by using serially diluted bovine sera in a negative serum (see Table 3). An overall evaluation of the results of this comparison and the results of the TG-ROC analyses above resulted in the selection of a cut-off value of S/P 15%.

### Reproducibility and variability of the ELISA

The reproducibility of the ELISA was tested by analyzing four different sera with S/P values ranging from 35 to 160 in an inter variation assay, on five different occasions. The mean results of the four sera tested in duplicates, gave a coefficient of variation (CV) of 5.0%. Analysis of the intra plate variation, including 12 replicates of the four sera on one plate, gave a mean CV of 4.5%. These data suggest that the in-house ELISA has the capacity to generate reproducible results with low variation.

### Comparison of the sensitivity and specificity between the in-house ELISA, VNT and a commercial ELISA

Table 2 shows the results of two-sided contingency comparison between the two ELISAs versus VNT in the analysis of bovine and ovine sera. Also, comparative analyses of 11 VNT positive and 100 negative caprine sera revealed a specificity of 100% for both ELISAs but higher sensitivity of the in-house ELISA compared to the commercial ELISA (11 positive compared to 9 positive, 1 doubtful and 1 negative). To compare the limits of detection between the in-house ELISA, the commercial ELISA and the VNT four serially diluted positive bovine sera were analyzed (see Table 3). The results showed a higher or equal sensitivity of the in-house ELISA compared to VNT. In comparison with the commercial ELISA, the in-house ELISA showed substantially higher sensitivity at analyzing two out of the four serially diluted sera (see Table 3, sera numbers 611 and 616). Analysis of the remaining two sera showed comparable sensitivity between the in-house ELISA and the commercial ELISA.

**Table 3:** **Comparison of sensitivity of the in-house Schmallenberg virus ELISA (A) and a commercial SBV ELISA (B) versus virus neutralization test (VNT)**

**Dilution of sera**	**Sera number**											
	**611**			**612**			**615**			**616**		
	**VNT**	**A**	**B**	**VNT**	**A**	**B**	**VNT**	**A**	**B**	**VNT**	**A**	**B**
1:1	**1:32**	**98**	**96**	**1:64**	**76**	**189**	**1:64**	**95**	**120**	**1:64**	**81**	**64**
1:4	**1:16**	**34**	53	**1:16**	**34**	**213**	**1:32**	**49**	**102**	**1:16**	**36**	25
1:8	**1:8**	**18**	34	**1:8**	**22**	**132**	**1:16**	**31**	**61**	**1:8**	**24**	21
1:16	1:4	9	17	1:4	**15**	**86**	1:4	**16**	35	<1:4	11	11
1:32	<1:4	3	8	<1:4	8	43	<1:4	7	16	1:4	6	−4
1:64	<1:4	3	4	<1:4	4	23	<1:4	5	9	<1:4	3	11

Four out of the positive sera from France (see Table 1); serially diluted 1:4 to 1:64 in negative serum were analyzed in accordance with the protocol of each test. The sera and sera dilutions where further diluted 1:100 in ELISA A, 1:10 in ELISA B and serially diluted 1:4 to 1:256 in VNT. Bold-figures indicate positive results, under-lined figures doubtful results. Thresholds: VNT titer ≥1:8 = positive. ELISA A; S/P% ≥15 = positive, ELISA B; S/P ≥50-60 = doubtful, >60 = positive.

## Discussion

The SBV is now established throughout Europe. The use of serological surveys makes large scale testing possible to map spread of infection and to better understand disease transmission dynamics. For serosurveillance and seromonitoring purposes in Sweden, an indirect ELISA was developed using whole SBV as antigen. In this work, we present the development and the evaluation of an indirect in-house ELISA to detect antibodies from cattle, sheep and goat against the full SB virus. Evaluation of the in-house ELISA was done by comparing results with VNT and a commercial ELISA based on a recombinant SBV nucleoprotein.

During the preparation of the ELISA SBV antigen from infected cell cultures it was found that the obtained cell material fraction contained considerable amount of viral proteins. This has previously been reported for virus belonging to the Simbu serogroup [18,19]. By utilizing the cell associated virus in combination with the non-cell-bound virus the antigen recovery was increased considerably with about 75%.

Triton x-100 was used for virus inactivation. This solvent/detergent works by disrupting the lipid layer of enveloped viruses. The method is effective, simple and rapid with a confirmed capacity to inactivate model enveloped viruses by >4 to >6 log after 30 min at 22°C [20,21]. The required amount of Triton x-100 for virus inactivation of SBV as confirmed after three passages on cell cultures was found to be 1.0% which is in compliance with previously reported experiments [21].

In assessing the cut-off value of the in-house ELISA, not only high sensitivity with a maximized specificity was taken into account, but also the varying distribution of the S/P values and the degree of separation between positive and negative sera of each group of cattle, sheep and goat sera. Based on TG-ROC analysis of positive and negative sera as well as comparative analysis of in-house ELISA and VNT of serially diluted bovine sera in negative bovine serum, the cut-off value of S/P =15 was finally selected giving an overall sensitivity of 99.19% and a specificity of 100%. The prevalence of both nucleocapside protein (N) and the glycoprotein (Gc) of the ELISA antigen were confirmed by western blot analysis. Along with the glycoprotein Gn, the Gc glycoprotein is involved in viral attachment and cell fusion and is considered to be the target for neutralizing antibodies [22-24]. The presence of both nucleocapside protein and Gc glycoprotein in the ELISA antigen may thus explain the similar sensitivity compared to VNT. Also, the broader spectrum of antigenic regions of the ELISA antigen may contribute to the higher sensitivity compared to a commercial ELISA which use a nucleocapside recombinant protein as antigen. The high performance of the in-house ELISA reported in this work was confirmed in a limited interlaboratory comparison of SBV antibody detection [25].

The conjugate, a monoclonal antibody against bovine IgG_1_, is developed and evaluated to detect cattle, sheep and goat antibodies in sera. Since IgG_1_ represent the major Ig class in milk throughout the lactation period, this conjugate was thought to be particularly useful in the detection of milk antibodies [26,27]. Further development and evaluation to optimize this in-house ELISA for analyzing bulk milk is in progress.

## Conclusion

An indirect ELISA was established with the use of whole virus as coating antigen and a monoclonal antibody for the detection of antibodies to SBV in bovine, ovine and caprine sera. The in-house ELISA showed good reproducibility and repeatability with high correlation to virus neutralization test. In a comparative study between the in-house ELISA, based on whole virus, and an ELISA based on recombinant nucleoprotein, the former showed a higher sensitivity indicating that the use of whole virus as antigen in this case, can be of advantage. The assay provides a rapid serological detection method that would be suitable for serosurveillance of SBV infection on a large scale.
